# Multiscale Analysis of the Microstructure and Stress Evolution in Cold Work Die Steel during Deep Cryogenic Treatment

**DOI:** 10.3390/ma11112122

**Published:** 2018-10-29

**Authors:** Junwan Li, Xin Cai, Yiwen Wang, Xiaochun Wu

**Affiliations:** 1 School of Materials Science and Engineering, Shanghai University, Shanghai 200444, China; ccxx218@163.com (X.C.); yiwenwang97@163.com (Y.W.); xcwu@staff.shu.edu.cn (X.W.); 2 State Key Laboratory of Advanced Special Steel, Shanghai University, Shanghai 200444, China

**Keywords:** deep cryogenic treatment, multiscale analysis, RVE, microstructure evolution, stress evolution

## Abstract

Through a combination of 3D representative volume element (RVE) and the metallo-thermo-mechanical coupling finite element (FE) analysis, a multiscale model was established to explore the localized characteristics of microstructure and stress evolution during deep cryogenic treatment (DCT). The results suggest that after cooling to near −160 °C, the largest intensity of martensite is formed, but the retained austenite cannot be eliminated completely until the end of DCT. The driving force for the precipitation of fine and uniform carbides during DCT is provided by the competition between the thermal and phase transformation stresses. Compared with the thermal stress, the phase transformation stress during DCT plays a more significant role. At the interface between retained austenite and martensite, a reduction of around 15.5% retained austenite even induces an obvious increase in the phase transformation stress about 1100 MPa. During DCT, the maximum effective stress in RVE even exceeds 1000 MPa, which may provide a required driving force for the precipitation of fine and homogeneously distributed carbide particles during DCT.

## 1. Introduction

As an extension of conventional heat treatment (CHT), deep cryogenic treatment (DCT) is widely accepted nowadays to enhance the physical and mechanical properties of materials [1,2]. It has received considerable attention in the last decade and has been employed in a wide range of manufacturing industries [3], including measuring tools, precision instrument and automobile industry, to improve the wear resistance [4,5], toughness [6,7], dimensional stability [8], fatigue life [9] and favorable residual stress condition of metal materials [10]. At present, Li et al. [11] found that by increasing the time and cycle number of DCT, the impact toughness and abrasive wear resistance of high-vanadium alloy steel can be further improved and the carbide contents continuously increase. Li et al. [12] examined the influences of cryogenic temperature, holding time and cooling rate on the high-temperature wear behavior of M2 steel for the process optimization and the understanding underlying mechanisms. Podgornik et al. [13] explored the impact of DCT on fracture toughness, wear resistance and load-carrying capacity of cold work tool steel. Wang et al. [14] proposed a novel quenching-partitioning-cryogenic-tempering (QPCT) treatment to further refine the microstructure and enhance the mechanical properties of a low-carbon Mn-Si-Cr alloyed steel. Pérez and Belzunce [15] considered that the application of DCT to H13 steel induces higher thermal stress and structural defect, produces a dispersed network of fine carbides after the subsequent tempering stages, and causes a significant improvement in the fracture toughness. Bensely et al. [16] examined the effect of CHT, shallow cryogenic treatment (SCT) and deep cryogenic treatment (DCT) on the fatigue properties of En 353 steel and revealed the different fracture mechanisms. Senthilkumar et al. [17] evaluated the influence of cryogenic treatment on the residual stress state in 4140 steel and suggested that DCT promotes a compressive residual stress. In the above investigations, the underlying mechanism of DCT can be explained by the phase transformation of retained austenite to martensite [18], the precipitation of fine and uniform carbides [19], and a more favorable residual stress state [17]. However, due to the limitation of experimental conditions, the underlying mechanisms of DCT are still not yet fully understood. For example, according to Gavriljuk et al. [20], the largest intensity of virgin, not aged martensite is formed at near −150 °C due a compromise between the driving force of transformation proportional to the decrease in temperature and the thermal activation needed for isothermal transformation kinetics, which plays a significant role in subsequent carbide precipitation during tempering. By observing the characterization of carbides during aging, Li et al. [21] points out that DCT increases the diffusion driving force of atoms of a low carbon steel which promotes the formation of fine carbides in the process of aging. However, these hypotheses or speculations have not been fully accepted because of the lack of direct evidence.

To realize the optimization of heat treatment process, some numerical models considering the coupling mechanisms existing among thermal, mechanical and metallurgical events have been developed to elucidate the details that might be inaccessible to experiments [22,23]. Recently, Lee et al. [24] proposed a new martensitic kinetics equation and combined the finite element model (FEM) simulation to explain the relationship between transformation kinetics and distortion during oil quenching of AISI 5120 steel. Yaakoubi et al. [25] elaborated a simulation of the thermo-metallurgical and mechanical coupling by using ABAQUS software and highlighted the metallurgical and mechanical behavior laws. Kim et al. [26] developed a finite element model considering the transformation plasticity to predict the deformation, carbon diffusion, phase fraction and hardness during the carburizing heat treatment of automotive annulus gear ring. However, compared with CHT, the modeling process of DCT is more complex, which must take into account the low temperature material properties and the nonlinear boundary condition of boiling heat transfer [27]. Li et al. [27,28] established a multi-physical field coupling numerical model to explore the evolution rule of retained austenite in Navy C-ring specimen during DCT. Although the multi-physical field coupled model can provide a reference for the optimization of DCT processes, it still cannot capture the physical detail of retained austenite and stress evolution in the realistic microstructure at the microscale. To solve this problem, micro-to-macro modeling has become one of the most popular forms of methods, including the representative volume element (RVE) method [29]. An RVE is supposed to represent all macroscopic properties of the micro heterogeneous material, which is developed based on the homogenization theory [30]. Presently, it has been widely adopted to understand the local deformation mechanics and mechanisms governing the macroscopic behavior of heterogeneous materials [31,32,33]. A combination of RVE and FEM is a valuable attempt to explore the characteristics and evolution regularity of microstructure and stress during DCT.

Based on the above motivations, the microstructure morphology of a cold work die steel SDC99 (Cr_8_Mo_2_SiV) after quenching treatment (QT) was evaluated in detail by using the scanning electron microscope (SEM), transmission electron microscope (TEM) and X-ray diffraction (XRD) analysis. A 3D RVE was reconstructed considering the features such as content, size, shape, spatial distribution of retained austenite and martensite after QT. Subsequently, through a combination of RVE and the metallo-thermo-mechanical coupling finite element (FE) analysis, a multiscale numerical model with the incorporation of a realistic material microstructure was established to reproduce the DCT process, capture the actual localized characteristics and evolution regularity of microstructure and stress, and provide a necessary theoretical basis for some understanding of DCT effects.

## 2. Experimental Procedures

A commercial cold work die steel SDC99 (Cr_8_Mo_2_SiV) was used in this investigation, which has a chemical composition of 0.91 C, 0.51 Si, 0.30 Mn, 8.60 Cr, 1.47 Mo, 0.3 V, 0.01 P, 0.0008 S and Fe balance (wt %). Due to its excellent hardenability and hardening capacity, it makes the phase transformation from austenite to martensite mainly occurred in the QT process. While, for the DCT process, as claimed in many published literatures [1,2], one of the main objectives was to eliminate or decrease the metastable retained austenite after QT by means of the martensite transformation at low temperature. Thus, the evolution and distribution of residual austenite during DCT was mainly considered in this study. 

To emphasize the non-simultaneity of cooling behavior and phase transformation during heat treatment, a unique C-ring specimen was selected to study the sensitivity of heat treatment conditions. The inside and outside diameters, thickness and gap width of the C-ring specimen were 15.9 mm, 25.4 mm, 19.1 mm and 6.4 mm, respectively. At first, the C-ring specimen was austenitized and homogenized for 2 h at 1040 °C under vacuum and subsequently water quenched. After QT, the specimen was immediately immersed in liquid nitrogen until its temperature cools down to −196 °C. The detail can be found in author’s early works [28]. After QT and DCT, both the scanning electron microscope (SEM, Zeiss, Supra 40, Jena, Germany) and the transmission electron microscope (TEM, JEOL, 2010F, Akishima, Japan) were used to examine the microstructural characterization of the C-ring specimen. The quantitative phase analysis of specimens after QT and DCT were performed by the X-ray diffraction (XRD) instrument (DLMax-2550, Rigaku, Osaka, Japan) at room temperature using Cr Kα (*λ* = 2.2909 Å) radiation. 

**Figure 1:**
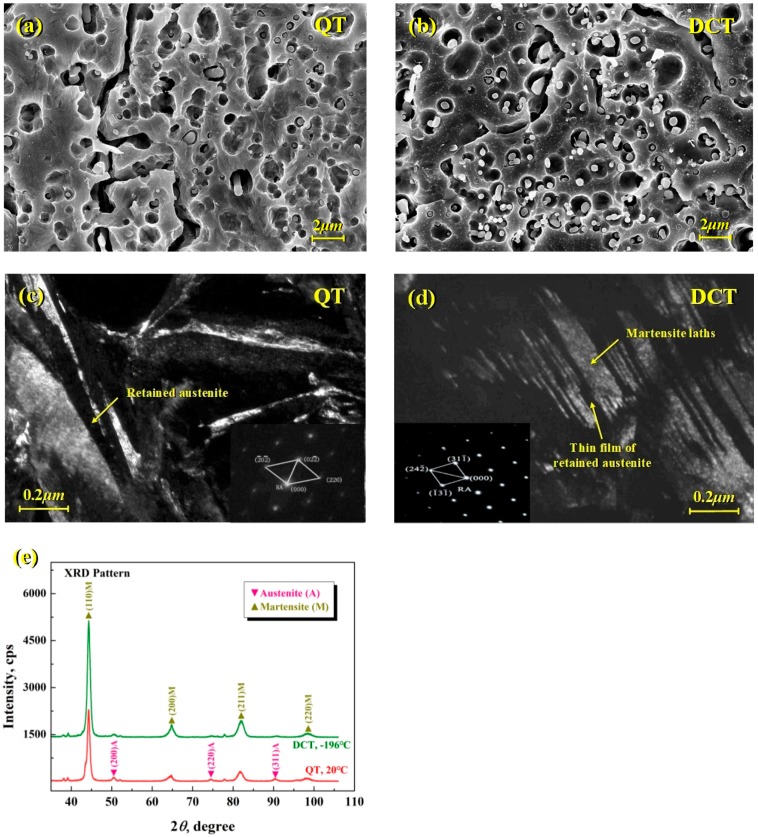
(**a**,**b**) scanning electron microscope (SEM) and (**c**,**d**) transmission electron microscope (TEM) photomicrographs, and (**e**) X-ray diffraction (XRD) pattern of cold work die steel SDC99 after quenching treatment (QT) and deep cryogenic treatment (DCT).

Figure 1 gives the SEM and TEM photomicrographs, and XRD pattern of cold work die steel SDC99 after QT and DCT. By comparison of the SEM photomicrographs illustrated in Figure 1a,b, it is clear that, after DCT, there are finer and homogeneously distributed of carbide particles precipitated on the matrix, which is one of the main reasons for the enhancement of mechanical properties of cold work die steel. As claimed in the pioneering works of DCT, some hypotheses or speculations suggests that it may be closely related with the driving force for transformation and the thermal activation [20,21], but they need more direct evidence to further validation. According to the TEM micrographs demonstrated in Figure 1c,d, after QT and DCT, there exist some obvious differences in the microstructure morphology, especially the metastable retained austenite. After QT, the microstructure of SDC99 steel mainly contains the lath and plate martensite and a large amount of retained austenite, which appears as block or a thick film (average about 100 nm) between the matrix grain boundaries. While, after DCT, apart from a uniform and fine carbide particle distribution on the matrix, the matrix is composed of the plate and lath martensite with a high density of dislocation and retained austenite with a morphology of thin film (about 10–30 nm). Figure 1e represents the X-ray diffraction pattern and calibration of SDC99 steel after QT and DCT. It can be seen that, after DCT, the (200), (220) and (311) austenite peaks exhibit a low intensity, which indicates the metastable retained austenite existed in the matrix after QT will continue to transform to martensite during DCT. After QT and DCT, the phase volume fractions of retained austenite and martensite are 17% and 83%, 2.8% and 97.2%, respectively. As mentioned in the literature on the DCT of tool steel [13], DCT can significantly decrease the amount of retained austenite and improve the microstructure homogeneity of steels, but it does not completely eliminate. Based on the above quantitative characterization of microstructure in C-ring specimen after QT such as content, size, shape and distribution of retained austenite and martensite, it can provide the basic data for the reconstruction of 3D RVE. Meanwhile, the volume fraction of retained austenite after DCT obtained from XRD analysis can also be used to verify the accuracy of multiscale simulation. 

## 3. Multiscale Numerical Procedure

**Figure 2:**
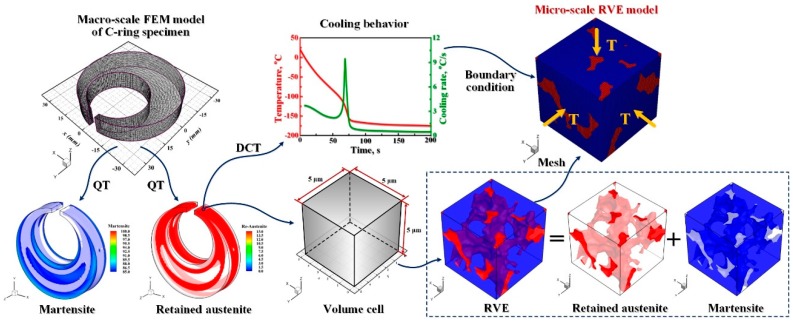
The multiscale modeling process from marco-to-micro scales of cold work die steel SDC99 C-ring specimen during DCT.

To capture the actual localized characteristics and evolution regularity of microstructure and stress in a realistic material microstructure, a multiscale model by a combination of the metallo-thermo-mechanical coupling FE analysis and RVE was established to reproduce the DCT process. The multiscale modeling process includes the macro-scale FEM modeling and the micro-scale RVE modeling, as shown in Figure 2. For the macro-scale FEM modeling, a metallo-thermo-mechanical coupling numerical model was built to explore the transient temperature and microstructure distribution in C-ring specimen during QT and DCT, especially the evolution rule of retained austenite. The main objective of the macro-scale FE simulation is to obtain the cooling behavior of C-ring specimen during DCT and provide the essential boundary condition of heat transfer for the micro-scale RVE analysis. The corresponding details of modeling can be found in author’s early works [27,28]. For the micro-scale RVE modeling, some important details should be pointed out: (1) according to author’s previous work [28], subjected to DCT, the metastable retained austenite in C-ring specimen principally congregates at the gap region of specimen. Due to the unique features of retained austenite evolution at the gap region of C-ring specimen during DCT, it was chosen to build RVE and impose the corresponding boundary condition of heat transfer, as plotted in Figure 2. (2) The spatial distribution of microstructure, especially the retained austenite, in RVE after QT was determined based on the quantitative characterization of microstructure at the gap region of C-ring specimen by SEM, TEM and XRD. (3) Considering the above microstructure features such as content, size, shape and distribution of retained austenite and martensite after QT, a 3D RVE with a size of 5 μm × 5 μm × 5 μm was reconstructed via an in-house numerical code developed by author. (4) Finally, combined the metallo-thermo-mechanical coupling model with RVE, the multiscale simulations of DCT were performed using the commercial FE code DEFORM-HT^®^ (Scientific Forming Technology Corporation, Columbus, OH, USA).

## 4. Results and Discussion

It is known that the influencing factors of DCT are extremely complicated, such as the minimum temperature, hold time, cooling and warming rate, which makes it very difficult to control in practice. For the underlying mechanism of DCT, regardless of the transformation from retained austenite to martensite, the precipitation and dispersion of fine carbides, or the more favorable residual stress distribution, it is closely associated with the evolution of microstructure and stress. During QT and DCT, the total strain rate ${\dot{\varepsilon }}_{ij}$ consists of the elastic ${\dot{\varepsilon }}_{ij}^{E}$, plastic ${\dot{\varepsilon }}_{ij}^{P}$ and thermo-metallurgical ${\dot{\varepsilon }}_{ij}^{\mathrm{TM}}$ parts, such that [27]:

(1) $${\dot{\varepsilon }}_{ij}={\dot{\varepsilon }}_{ij}^{E}+{\dot{\varepsilon }}_{ij}^{P}+{\dot{\varepsilon }}_{ij}^{\mathrm{TM}}$$

where, the thermo-metallurgical ${\dot{\varepsilon }}_{ij}^{\mathrm{TM}}$ one can decomposed into the thermal ${\dot{\varepsilon }}_{ij}^{\mathrm{Th}}$, dilatational phase transformation ${\dot{\varepsilon }}_{ij}^{\mathrm{Ph}}$ and transformation plasticity ${\dot{\varepsilon }}_{ij}^{\mathrm{Trp}}$ parts as follows:

(2) $${\dot{\varepsilon }}_{ij}^{\mathrm{TM}}={\dot{\varepsilon }}_{ij}^{\mathrm{Th}}+{\dot{\varepsilon }}_{ij}^{\mathrm{Ph}}+{\dot{\varepsilon }}_{ij}^{\mathrm{Trp}}$$

Here, due to the absence of applied load, the transformation plasticity one can be disregarded in this study. For the thermal strain rate, it relates to the instantaneous thermal expansion coefficient *α_k_* and the temperature change, which can be described as [27]:

(3) $${\dot{\varepsilon }}_{ij}^{\mathrm{Th}}=\alpha_{k}\delta_{ij}\frac{dT}{dt}$$

while, for the dilatational phase transformation strain rate, it is the strain rate due to the structural dilatation, which can be calculated by [27]

(4) $${\dot{\varepsilon }}_{ij}^{\mathrm{Ph}}=\Delta_{k}\frac{d\xi_{k}}{dt}$$

where Δ*_k_* represents the structural dilation due to the decomposition of austenite to the *k*th microstructural constituent. *ξ_k_* is the volume fraction of phases.

**Figure 3:**
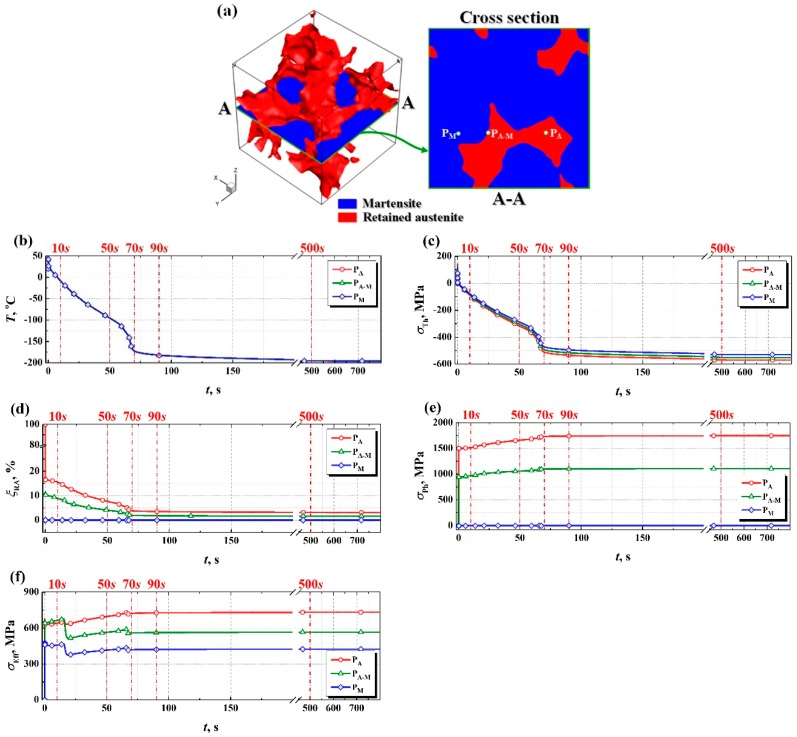
(**a**) Tracking points on the central cross section of representative volume element (RVE) and the evolution of (**b**) temperature, (**c**) thermal stress, (**d**) volume friction of retained austenite, (**e**) phase transformation stress and (**f**) effective stress at tracking points on the cross section of RVE during DCT.

For the purpose of comparison, on the central cross section of RVE, three different tracking points are selected to monitor the time-dependent phase transformation and stress evolution during DCT, as labeled with P_A_, P_M_ and P_A-M_ in Figure 3a. The corresponding positions of three tracking points at the initial of DCT are located in retained austenite, martensite and at the interface between retained austenite and martensite, respectively. Figure 3b represents the time-dependent temperature (*T*) curves at tracking points P_A_, P_M_ and P_A-M_ during DCT. Due to the relatively small size of RVE, under the given boundary condition of heat transfer, the cooling curves of tracking points P_A_, P_M_ and P_A-M_ during DCT exhibit an almost identical trend. As the cooling time of DCT is prolonged, the temperature of tracking point gradually decreases and after cooling to −160 °C (about 70 s) of DCT, the cooling rate achieves its maximum value of around 14 °C/s. Subsequently, the temperature tends to an equilibrium value about −196 °C. According to the cooling behavior of different tracking points during DCT illustrated in Figure 3b, combined with Equation (3), the time-dependent thermal stresses (*σ*_Th_) at tracking points P_A_, P_M_ and P_A-M_ can also be determined, as shown in Figure 3c. Although there is almost no difference in the cooling behavior of all tracking points during DCT, the difference of thermal stress at different tracking points cannot be neglected due to the different thermal expansion coefficients of retained austenite and martensite. Corresponding to the maximum cooling rate occurred at −160 °C of DCT, the thermal stresses of all tracking points also experience a sudden increase. After DCT, the thermal stresses at tracking points P_A_, P_M_ and P_A-M_ are approximately −570 MPa, −530 MPa and −553 MPa, respectively. However, it is noteworthy that the thermal stresses at different tracking points during DCT are always negative, which means that RVE is in a state of thermal contraction and undergoes a thermal contraction stress. Figure 3d displays the retained austenite (*ξ*_RA_) evolution at tracking points P_A_, P_M_ and P_A-M_ during DCT. For the tracking point P_M_, because it located in the martensite phase, there is no phase transition even at very low temperature. While, for the tracking points P_A_ and P_A-M_, the retained austenite will continue to convert to martensite until the temperature reaches about −160 °C (about 70 s) of DCT. At the end of DCT, the volume fractions of retained austenite at tracking points P_A_ and P_A-M_ are about 3.05% and 1.53%, respectively, which are almost identical with the XRD measurements and this transformation from retained austenite to martensite is still incomplete as mentioned in References [7,28]. Due to the structural dilation induced by the transformation from retained austenite to martensite during DCT, it will inevitably give rise to the phase transformation stress (*σ*_Ph_). Based on Equation (4), one can get the time-dependent phase transformation stress (*σ*_Ph_) at different tracking points, as demonstrated in Figure 3e, which is proportional to the amount of retained austenite transformed into martensite during DCT. For tracking point P_M_, its phase transformation stress is zero. Apart from the tracking point P_M_, with the increase of cooling time, the phase transformation stresses at both tracking points P_A_ and P_A-M_ during DCT will increase gradually, and then when their temperatures drop to about −160 °C, these phase transformation stresses achieve a steady state. At the end of DCT, the phase transformation stresses inside the retained austenite grain, namely point P_A_ is almost 1750 MPa. While, at the interface between retained austenite and martensite, namely point P_A-M_, it also exceeds 1100 MPa. However, it should be emphasized that the phase transformation stresses at different tracking points during DCT are always positive, which implies that RVE is in a state of volumetric expansion and suffers from a volumetric expansion stress. Compared with the thermal stresses, the phase transformation stresses at different tracking points during DCT are obviously larger, especially at the point P_A_, which is even 3 times higher than its thermal stress. In addition, by means of the Von Mises yield criterion and the flow stress model [27], the effective stress (*σ*_Eff_) at different tracking points during DCT can also be evaluated, as presented in Figure 3f. At the beginning of DCT, there is an abrupt drop in the effective stress at tracking points P_A-M_ and P_M_, which may be related to stress relaxation. After cooling to about −160 °C, the effective stresses at tracking points P_A_, P_M_ and P_A-M_ approach to the steady state and their values are about 720 MPa, 415 MPa and 560 MPa, respectively.

According to the above analysis of microstructure and stress evolution in RVE demonstrated in Figure 3, it can be found: (i) after cooling to near −160 °C of DCT, the cooling rate achieves its maximum value and the largest intensity of martensite is formed, which is consistent with Gavriljuk et al. [20], but the retained austenite has not been eliminated completely. (ii) As suggested in Reference [20], the required driving force for the precipitation of fine and uniform carbides is provided by the competition between the thermal and phase transformation stresses during DCT. When the temperature drops to near −160 °C, both the thermal and phase transformation stresses achieve the steady state. However, compared with the thermal stress, the phase transformation stress during DCT plays a more significant role. For example, at the interface between retained austenite and martensite, a reduction of around 15.5% retained austenite even induces an obvious increase in the phase transformation stress about 1100 MPa. (iii) During DCT, the maximum value of effective stress at different tracking points is even more than 720 MPa. The effective stress level is so high, particularly in the retained austenite and at the interface between retained austenite and martensite, that it can provide a potential driving force for the formation of fine and uniformly dispersed carbides during DCT.

**Figure 4:**
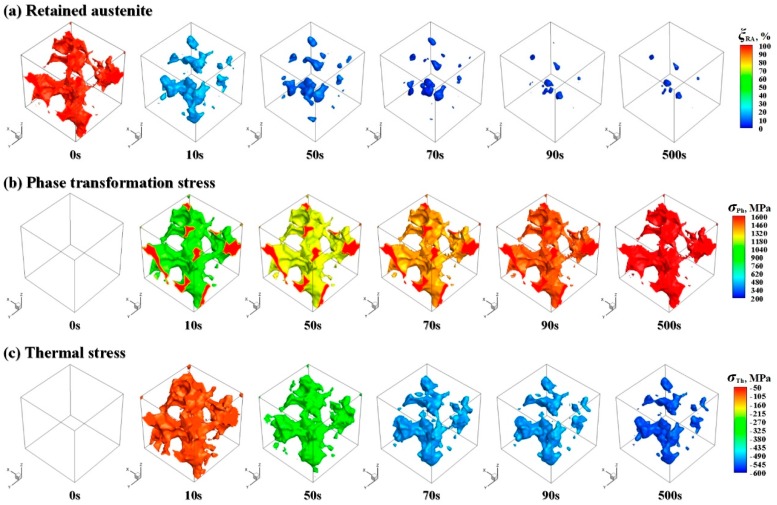

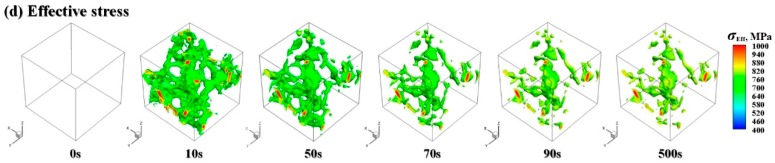
Spatial distribution and evolution of (**a**) retained austenite, (**b**) phase transformation stress, (**c**) thermal stress and (**d**) effective stress in RVE during DCT.

In order to visually observe the localized characteristics of microstructure and stress evolution, Figure 4 shows the spatial distribution of retained austenite, phase transformation stress, thermal stress and effective stress in RVE at different time during DCT. Actually, during DCT, the interface between retained austenite and martensite in RVE is constantly changing with the increase of cooling time. For the convenience of comparison, both the regions of retained austenite and its interface are highlighted by color. Figure 4a gives the detail of retained austenite evolution in RVE during DCT. Since the non-diffusion-controlled martensitic transformation only depends on temperature and is not controlled by temperature history, there is a rapid reduction of retained austenite content in RVE at the initial stage of DCT. After cooling to −160 °C (about 70 s) of DCT, most of the retained austenite in RVE has been transformed into martensite, but it will not be completely eliminated. Until the end of DCT, there still exist almost 3% of retained austenite in RVE, which is in good agreement with the XRD result, as its final spatial distribution represented at 500 s in Figure 4a. Figure 4b is the spatial distribution of phase transformation stress in RVE at different time during DCT. With the decrease of retained austenite, the phase transformation stress in RVE presents an accumulation phenomenon. At the early stage of DCT, it increases significantly and then gradually becomes stable after about 70 s of DCT. After DCT, the mean phase transformation stresses inside retained austenite grain and at the interface between retained austenite and martensite in RVE are almost up to 1500 MPa, as plotted at 500 s in Figure 4b. Figure 4c represents the thermal stress contour in RVE at different time during DCT. Different from phase transformation stress, the value of thermal stress during DCT are always negative and exhibits a compressive stress. The amplitude of thermal stress is significantly smaller and only about 1/3 of the phase transformation stress. Subjected to DCT, the mean thermal stress in RVE is approximately −550 MPa. Figure 4d demonstrates the evolution regularity of effective stress in RVE during DCT. As can be seen from this figure, after cooling to about −160 °C (about 70 s), the effective stress at the interface between retained austenite and martensite is always maintained above 550 MPa or even higher during DCT. While, in the retained austenite grain, its maximum even exceeds 1000 MPa, which may provide a required driving force for the precipitation of fine carbide particles during DCT as observed in the SEM photomicrographs.

## 5. Conclusions

In this investigation, through a combination of 3D RVE and the metallo-thermo-mechanical coupling FE analysis, a multiscale model incorporating the realistic material microstructure of cold work die steel SDC99 was built to explore the localized characteristics of microstructure and stress evolution during DCT. The following conclusions are presented:

1. After cooling to near −160 °C of DCT, the largest intensity of martensite is formed, but the retained austenite has not been eliminated completely until the end of DCT and there still exist almost 3% of retained austenite in RVE.
2. The driving force for the precipitation of fine and uniform carbides during DCT is provided by the competition between the thermal and phase transformation stresses. After DCT, the mean phase transformation stresses inside retained austenite grain and at the interface between retained austenite and martensite in RVE are almost up to 1500 MPa. While, the amplitude of thermal stress is significantly smaller and only about 1/3 of the phase transformation stress. Compared with the thermal stress, the phase transformation stress during DCT plays a more significant role.
3. During DCT, the maximum value of effective stress in RVE even exceeds 1000 MPa, which may provide a required driving force for the precipitation of fine carbide particles during DCT. The above quantitative findings can provide valuable insights for better understanding of the underlying mechanism of DCT and support its further quantitative analysis.

## References

[1] Kalsi N.S., Sehgal R., Sharma V.S. (2010). Cryogenic treatment of tool materials: A review. Mater. Manuf. Process..

[2] Gill S.S., Singh H., Singh R., Singh J. (2010). Cryoprocessing of cutting tool materials—A review. Int. J. Adv. Manuf. Technol..

[3] Gu K.X., Zhao B., Weng Z.J., Wang K.K., Cai H.K., Wang J.J. (2018). Microstructure evolution in metastable β titanium alloy subjected to deep cryogenic treatment. Mater. Sci. Eng. A.

[4] Das D., Dutta A.K., Toppo V., Ray K.K. (2007). Effect of deep cryogenic treatment on the carbide precipitation and tribological behavior of D2 steel. J. Mater. Manuf. Process..

[5] Wang K.K., Tan Z.L., Gu K.X., Gao B., Gao G.H., Misra R.D.K., Bai B.Z. (2017). Effect of deep cryogenic treatment on structure-property relationship in an ultrahigh strength Mn-Si-Cr bainite/martensite multiphase rail steel. Mater. Sci. Eng. A.

[6] Li S.H., Xie Y.Z., Wu X.C. (2010). Hardness and toughness investigations of deep cryogenic treated cold work die steel. Cryogenics.

[7] Ptačinová J., Sedlická V., Hudáková M., Dlouhý I., Jurči P. (2017). Microstructure-toughness relationships in sub-zero treated and tempered Vanadis 6 steel compared to conventional treatment. Mater. Sci. Eng. A.

[8] Surberg C.H., Stratton P., Lingenhöle K. (2008). The effect of some heat treatment parameters on the dimensional stability of AISI D2. Cryogenics.

[9] Nazarian H., Krol M., Pawlyta M., Vahdat S.E. (2018). Effect of sub-zero treatment on fatigue strength of aluminum 2024. Mater. Sci. Eng. A.

[10] Araghchi M., Mansouri H., Vafaei R., Guo Y.N. (2017). A novel cryogenic treatment for reduction of residual stresses in 2024 aluminum alloy. Mater. Sci. Eng. A.

[11] Li H.Z., Tong W.P., Cui J.J., Zhang H., Chen L.Q., Zuo L. (2016). The influence of deep cryogenic treatment on the properties of high-vanadium alloy steel. Mater. Sci. Eng. A.

[12] Li J.J., Yan X.G., Liang X.Y., Guo H., Li D.Y. (2017). Influence of different cryogenic treatments on high-temperature wear behavior of M2 steel. Wear.

[13] Podgornik B., Paulin I., Zajec B., Jacobson S., Leskovšek V. (2016). Deep cryogenic treatment of tool steels. J. Mater. Process. Technol..

[14] Wang K.K., Gu K.X., Miao J.H., Weng Z.J., Wang J.J., Tan Z.L., Bai B.Z. (2018). Toughening optimization on a low carbon steel by a novel Quenching-Partitioning-Cryogenic-Tempering treatment. Mater. Sci. Eng. A.

[15] Pérez M., Belzunce F.J. (2015). The effect of deep cryogenic treatments on the mechanical properties of an AISI H13 steel. Mater. Sci. Eng. A.

[16] Bensely A., Shyamala L., Harish S., Mohan Lal D., Nagarajan G., Junik K., Rajadurai A. (2009). Fatigue behavior and fracture mechanism of cryogenically treated En 353 steel. Mater. Des..

[17] Senthilkumar D., Rajendran I., Pellizzari M., Siiriainen J. (2011). Influence of shallow and deep cryogenic treatment on the residual state of stress of 4140 steel. J. Mater. Process. Technol..

[18] Zhirafar S., Rezaeian A., Pugh M. (2007). Effect of cryogenic treatment on the mechanical properties of 4340 steel. J. Mater. Process. Technol..

[19] Das D., Dutta A.K., Ray K.K. (2009). On the refinement of carbide precipitates by cryotreatment in AISI D2 steel. Philos. Mag..

[20] Gavriljuk V.G., Theisen W., Sirosh V.V., Polshin E.V., Kortmann A., Mogilny G.S., Petrov Y.N., Tarusin Y.V. (2013). Low-temperature martensitic transformation in tool steels in relation to their deep cryogenic treatment. Acta Mater..

[21] Li S.H., Xiao M.G., Ye G.M., Zhao K.Y., Yang M.S. (2018). Effects of deep cryogenic treatment on microstructural evolution and alloy phases precipitation of a new low carbon martensitic stainless bearing steel during aging. Mater. Sci. Eng. A.

[22] Li H.P., Zhao G.Q., Niu S.T., Huang C.Z. (2007). FEM simulation of quenching process and experimental verification of simulation results. Mater. Sci. Eng. A.

[23] Jung M., Kang M., Lee Y.K. (2012). Finite-element simulation of quenching incorporating improved transformation kinetics in a plain medium-carbon steel. Acta Mater..

[24] Lee S.J., Lee Y.K. (2008). Finite element simulation of quench distortion in a low-alloy steel incorporating transformation kinetics. Acta Mater..

[25] Yaakoubi M., Kchaou M., Dammak F. (2013). Simulation of the thermomechanical and metallurgical behavior of steels by using ABAQUS software. Comput. Mater. Sci..

[26] Kim D.W., Cho H.H., Lee W.B., Cho K.T., Cho Y.G., Kim S.J., Han H.N. (2016). A finite element simulation for carburizing heat treatment of automotive gear ring incorporating transformation plasticity. Mater. Des..

[27] Li J.W., Tang L.L., Li S.H., Wu X.C. (2013). Finite element simulation of deep cryogenic treatment incorporating transformation kinetics. Mater. Des..

[28] Li J.W., Feng Y., Tang L.L., Wu X.C. (2013). FEM prediction of retained austenite evolution in cold work die steel during deep cryogenic treatment. Mater. Lett..

[29] Bargmann S., Klusemann B., Markmann J., Schnabel J.E., Schneider K., Soyarslan C., Wilmers J. (2018). Generation of 3D representative volume elements for heterogeneous materials: A review. Prog. Mater. Sci..

[30] Drugan W.J., Willis J.R. (1996). A micromechanics-based nonlocal constitutive equation and estimates of representative volume element size for elastic composites. J. Mech. Phys. Solids.

[31] Bong H.J., Lim H., Lee M.G., Fullwood D.T., Homer E.R., Wagoner R.H. (2017). An RVE procedure for micromechanical prediction of mechanical behavior of dual-phase steel. Mater. Sci. Eng. A.

[32] Berisha B., Raemy C., Becker C., Gorji M., Hora P. (2015). Multiscale modeling of failure initiation in a ferritic-pearlitic steel. Acta Mater..

[33] Pinz M., Weber G., Lenthe W.C., Uchic M.D., Pollock T.M., Ghosh S. (2018). Microstructure and property based statistically equivalent RVEs for intragranular γ−γ’ microstructures of Ni-based superalloys. Acta Mater..

